# Co-occurring functional neurological disorder and autism: an exploratory study of comorbidities in a retrospective cohort study using TriNetX

**DOI:** 10.1007/s00415-025-13385-6

**Published:** 2025-09-25

**Authors:** Lily Smythe, Livia Asan, Timothy R. Nicholson, Francesca Happé, Mark J. Edwards

**Affiliations:** 1 Social, Genetic and Developmental Psychiatry Centre, Institute of Psychiatry, Psychology and Neuroscience, King’s College London, London, UK; 2 Department of Neurology, Centre for Translational and Behavioural Neurosciences, University Hospital Essen, University of Duisburg-Essen, Essen, Germany; 3 Neuropsychiatry Research and Education Group, Institute of Psychiatry, Psychology and Neuroscience, King’s College London, London, UK; 4 Department of Basic and Clinical Neuroscience, Institute of Psychiatry, Psychology and Neuroscience, King’s College London, London, UK; 5 South London and Maudsley NHS Trust, London, UK

**Keywords:** Autism, Functional neurological disorder, Functional seizures, Attention-deficit/hyperactivity disorder

## Abstract

**Background:**

Functional Neurological Disorder (FND) encompasses motor, cognitive, and sensory symptoms resulting from disruptions in brain-body communication. Emerging research suggests a higher-than-expected occurrence of autism in FND, potentially due to shared cognitive mechanisms and overlapping comorbidities. However, large-scale characterisation of this dual-diagnosis is lacking.

**Methods:**

Using de-identified health records from the TriNetX research network, we identified children and adults with both FND and autism (‘FND + Autism’), comparing them to individuals with FND only (‘FND-only’) and autism only (‘Autism-only’). We examined psychiatric comorbidities (e.g. mood, anxiety, post-traumatic stress disorder, personality disorders, obsessive–compulsive disorder), intellectual disability and ADHD.

**Results:**

Of 220,312 individuals with an FND diagnosis, and 674,971 individuals with an autism diagnosis, 5,152 (2.3% of FND, 0.76% of autism) had both FND and autism. The rates of autism were therefore 6 times higher in FND compared to the base rates of the TriNetX population. Most were diagnosed with autism before FND, with over one-third diagnosed in childhood. Functional seizures were the most common FND subtype, and were more frequent in FND + Autism than FND-only (adults: 52% vs. 44%; children: 47% vs. 42%). Comorbidity across all psychiatric conditions was significantly higher in FND + Autism compared to both comparison groups. ADHD was particularly elevated in FND + Autism (adults: 50% vs. 13% FND-only, 36% Autism-only; children: 64% vs. 21% FND-only, 41% Autism-only).

**Conclusions:**

This study presents the largest dataset to date characterising individuals with co-occurring FND and autism. Findings are consistent with previous findings of higher rates of autism in people with FND and reveal a potentially distinct clinical profile, marked by elevated rates of ADHD and psychiatric comorbidities, and increased occurrence of functional seizures compared to FND- or Autism-only groups. Recognising this overlap may improve diagnosis, clinical care, and understanding of mechanisms underlying the co-occurrence of FND and autism.

**Supplementary Information:**

The online version contains supplementary material available at 10.1007/s00415-025-13385-6.

## Background

Functional neurological disorder (FND) describes motor, cognitive, and sensory symptoms (e.g., weakness, tremors, seizures, or memory difficulties) thought to arise from disrupted brain control over voluntary actions and bodily signals—particularly those typically under conscious control. FND shows a male-to-female ratio of approximately 1:3 [1].

There is growing recognition of FND co-occurring with autism, a neurodevelopmental condition characterised by social communication difficulties, rigid/repetitive behaviours and interests, and sensory atypicalities [2]. A recent meta-analysis of 24 studies involving over 11,000 participants estimated the autism prevalence in the FND population at ~ 10% [3], compared to an estimated autism prevalence of 1–2% globally [4].

Autistic traits also appear elevated in individuals with FND. One study found 69% of FND patients scoring above the suggested clinically significant cut-off for likely autism on the Adult Autism Subthreshold Spectrum self-report questionnaire, and a further 21% with high traits, while 24% reported a 1st-degree relative with a formal autism diagnosis [5].

As autism is a lifelong condition, there are important questions regarding how its neurobiology and psychosocial impact may increase vulnerability to developing FND. Several shared mechanisms have been proposed, including interoceptive deficits, altered attention, and alexithymia and emotion regulation difficulties (e.g. [6, 7]). Studies have particularly noted elevated autism rates among individuals with functional seizures, especially in children [8].

Psychiatric comorbidities are common in both autism and FND, with over half of individuals in each group affected [3]. There is notable overlap in conditions such as anxiety, posttraumatic stress disorder (PTSD), obsessive–compulsive disorder (OCD), and mood disorders (e.g., [9, 10]). Personality disorders, especially borderline personality disorder (BPD), have been linked to both conditions, with autism potentially misdiagnosed as BPD in some individuals [11]. Obsessive–compulsive personality disorder (OCPD) has also been linked to FND [12].

These comorbidities are clinically relevant, as the accumulation of psychological stressors- rather than discrete trauma- may contribute FND onset [10]. It is therefore possible that the interplay of shared biological vulnerabilities and the psychosocial burden of undiagnosed or unsupported autism [13] may further elevate FND risk.

Autism frequently co-occurs with attention-deficit/hyperactivity disorder (ADHD), with a recent meta-analysis estimating lifetime ADHD prevalence in autism at 40% [14]. While autism has received increasing attention in FND research, ADHD remains unexplored. Given autism’s elevated prevalence in FND and its frequent co-occurrence with ADHD, it is plausible that ADHD is also more prevalent in FND, especially when co-occurring with autism. Intellectual disability is another common co-occurrence in autism [15] and is relevant given the added clinical complexity of individuals with overlapping FND, autism, and intellectual disability.

Existing literature on the FND and autism co-occurrence is limited by small sample sizes, differing methodologies, and a narrow focus on specific FND subtypes (e.g. functional seizures). Broader, large-scale characterisation could identify patterns of comorbidity and inform how diagnostic and treatment services might be tailored to better meet the needs of individuals with both conditions.

Against this background, the present study aimed to characterise the clinical profile of individuals with co-occurring FND and autism, compared to those with either diagnosis alone. Given increasing recognition of shared vulnerabilities- particularly in psychiatric comorbidities- we examined the prevalence of mood disorders, anxiety, PTSD, OCD, and personality disorders across the three groups using data from a large-scale electronic health records network (TriNetX). ADHD and intellectual disability were also included due to their relevance in autism and implications for clinical complexity. We explored whether the distribution of FND subtypes differed across groups, with particular interest in functional seizures given their reported association with autism. We anticipated that the dual-diagnosed group would show higher rates of comorbidities, including ADHD, compared to individuals with FND and autism alone.

## Methods

### TriNetX research network

TriNetX is a global health resource network providing access to electronic medical records (e.g. diagnoses, procedures) from approximately 176 million patients across 141 healthcare organisations (HCOs). Data for this study were accessed on 23rd April 2025. Diagnoses are recorded using the International Classification of Diseases, Tenth Revision, Clinical Modification (ICD-10-CM; [16]).

As this study involved secondary analysis of an existing de-identified dataset, ethical approval was not required, in line with King’s College London Research Ethics Committee guidance.

### Study population

Patients with co-occurring autism and FND (hereafter ‘FND + Autism’) were identified using ICD-10-CM criteria (see Table 1). This group was compared to two cohorts: patients with FND but no autism (‘FND-only’) and those with autism but no FND (‘Autism-only’). These comparison groups enabled the characterisation of the FND + Autism population and assessment of comorbidity patterns.

**Table 1:** ICD-10 codes used to define FND and autism

Group	Inclusion
FND (F44*)	F44.4—Conversion disorder with motor symptoms F44.5—Conversion disorder with seizures F44.6—Conversion disorder with sensory symptoms or deficit F44.7—Conversion disorder with mixed symptom presentation F44.8—Other dissociative and conversion disorder F44.9—Unspecified conversion disorder
Autism (F84**)	F84.0—Autistic disorder F84.5—Asperger’s syndrome

*The category including FNDs in ICD-10 is ‘Dissociative and conversion disorders’

**The category including autism in the ICD-10 is ‘Pervasive developmental disorders’

### Inclusion of comorbidities

Comorbidities were selected from ICD-10-CM mental and behavioural disorders (F category), based on prior associations with FND and/or autism. Diagnoses in TriNetX are cumulative, including all diagnoses recorded in a patient’s medical record up to the point of data extraction. We cannot be certain that revised diagnoses lead to the removal of previous diagnoses. The ICD-10-CM codes used in the final analysis are listed in Table S2 in the supplementary information.

Anxiety disorders were defined using the F41 category (‘Other anxiety disorders’), including panic disorder, generalised anxiety disorder, and anxiety disorder unspecified. Mood disorders were defined using the F30-F39 categories (‘Mood [affective] disorders’), encompassing bipolar disorder, depressive episodes, and major depressive disorders. To minimise the number of statistical comparisons and reduce type 1 error categories were collapsed to represent broad groupings of anxiety and mood disorders.

‘Specific personality disorders’ (F60) encompasses ‘Emotionally unstable personality disorder’, and ‘Anankastic personality disorder’ amongst others. These subtypes will henceforth be referred to by their more common names, ‘Borderline personality disorder’ (BPD), and ‘Obsessive–compulsive personality disorder’ (OCPD) respectively.

### Statistical analysis

For each comorbidity, total numbers and percentages of adult and child patients across the three groups were reported. Chi-square tests assessed group differences in comorbidity rates, and odds ratios are reported. Effect size was measured using the phi coefficient (ϕ), interpreted as small (0.1), medium (0.3), or large (0.5) based on Cohen’s criteria [20]. All statistical analyses were conducted using RStudio.

## Results

### Clinical characteristics of patients

Out of the TriNetX population of 176 million individuals, 674,971 (0.38%) had a diagnosis of autism, while 220,312 (0.12%) had a diagnosis of FND. Among those with FND, 5,152 individuals (2.3%) also had a co-occurring diagnosis of autism. A diagnosis of autism was 5.7 times more common in those with a diagnosis of FND than in the general population included in the TriNetX database.

FND and autism showed distinct age distributions: autism diagnoses peaked in childhood and declined after age 30, while FND was rare in childhood but more evenly distributed from ages 20 to 60. Consequently, most patients with FND + Autism clustered between ages 18–24. To account for these patterns, potential differences in childhood vs. adult-onset FND [18], and trends in adolescent [19] and late-onset [20] FND, we divided the sample into child (10–18 years) and adult (19–50 years) cohorts. This also addressed the very small number of dual diagnoses over age 50, likely due to the well-evidenced underdiagnosis of autism in older adults [21].

Additionally, FND and autism differed by sex: 73% of the autism group were males, while 69% of the FND group were females. Therefore, sex-stratified analyses were performed for both age groups to assess sex-related effects.

**Fig. 1:**
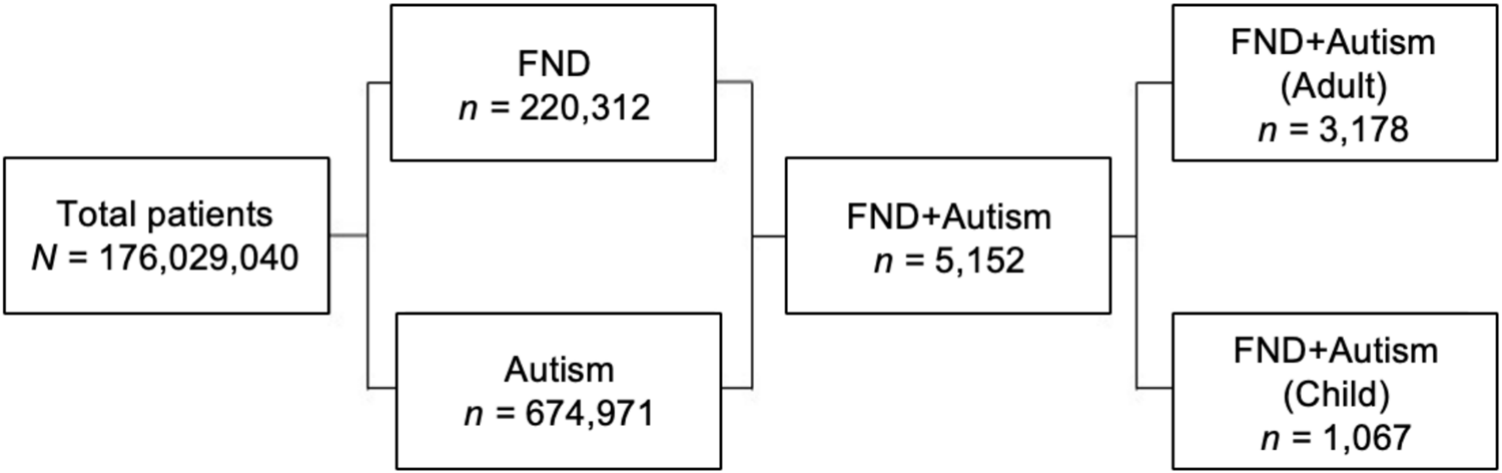
Flowchart of patient group sizes for patients identified with co-occurring FND+Autism in the TriNetX database

After applying age truncation (10–50 years), 4245 people with co-occurring FND and autism remained: 1067 children and 3178 adults. Figure 1 shows a flow diagram illustrating group sizes.

Demographic characteristics for adult and child patients across the three diagnostic groups are summarised in Table 2. Patients in the FND-only group for adults and children were predominantly female, while those in the Autism-only group were predominantly male. This sex disparity reduced in the FND + Autism group, with a more even split, particularly in the child group.

**Table 2:** Patient demographics for the adult and child cohorts

	Adult (*N* = 324,169)			Child (*N* = 230,115)		
	FND + autism	FND-only	Autism-only^†^	FND + autism	FND-only	Autism-only^†^
*n*	3178	85,746	235,245	1067	11,155	217,893
HCOs (/141)	83	140	137	73	123	131
Sex %						
Female	57.75	72.82	26.04	51.45	70.91	23.45
Male	39.55	25.58	71.87	48.17	28.69	75.46
Unknown	2.71	1.60	2.09	0.94	0.40	1.09
Mean age (SD) years	28 (8)	34 (9)	27 (7)	15 (2)	16 (2)	14 (3)
Race %						
White	72.78	59.58	64.22	64.67	53.61	54.86
Black or African	8.46	14.71	10.17	12.74	16.84	12.52
Asian	1.86	2.54	3.31	4.78	4.23	4.13
American Indian	0.82	0.70	0.46	0.94	0.48	0.57
Native Hawaiian	0.44	0.66	0.43	0.94	0.36	0.44
Other race	1.86	2.54	3.31	4.87	4.23	4.13
Unknown	12.49	18.19	16.77	12.00	18.48	19.39

*Note.* SD = Standard deviation; ^†^ The total number of patients included in the final analyses differed in the Autism-only group due to TriNetX capping large cohort results to approximately 10,000 patients per Healthcare Organisation (HCO). Total adult Autism-only prior to capping was 236,166; and total child Autism-only prior to capping was 232,761

In response to reviewer queries regarding the representativeness of the base autism prevalence in TriNetX, we sought to explore the rates of autism in children and adults with FND compared to the background population. Due to the live nature of the TriNetX database, between the initial and updated database pulls, the TriNetX population increased by ~ 2%, resulting in small shifts in absolute numbers but no change in overall trends. In the updated analysis, background autism prevalence was 0.23% in adults and 1.8% in children. Among individuals with FND, autism was present in 2.2% of adults and 9.6% of children. This equated to autism being 5.7 times more common in FND overall (2.55% vs. 0.45%), 5.3 times more common in children with FND (9.6% vs. 1.8%), and 9.7 times more common in adults with FND (2.2% vs. 0.23%).

### FND and autism diagnosis

**Fig. 2:**
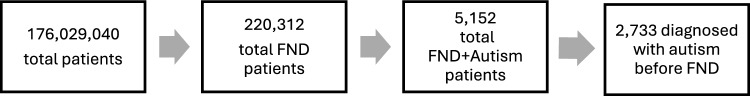
Characteristics of patients with FND+Autism, illustrating the number of patients diagnosed with autism prior to their FND diagnosis, and in childhood

5,152 patients with FND + Autism were identified, representing 2.3% of FND patients and 0.76% of autism patients (Fig. 2). Among them, 53% received an autism diagnosis before FND, and 34% were diagnosed with autism before age 18 years.

### FND subtypes

**Fig. 3:**
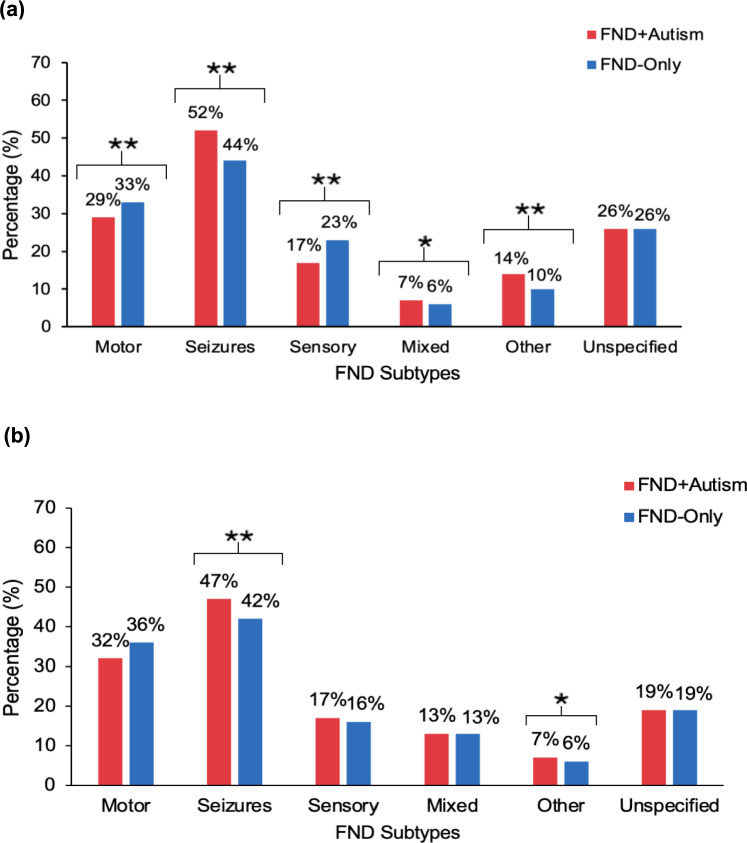
Percentage of FND subtypes in FND+Autism and FND-only groups in (a) adults and (b) children.Note. * = *p* <.05; ** = *p* < 0.001

Figure 3 and Supplementary Table S4 (see supplementary information) detail the distribution and analysis of FND subtypes in adult and child cohorts. Seizures (F44.5) were the most common F44 subtype in both groups, with FND + Autism adults exhibiting a significantly higher rate of seizures (52%) compared to FND-only adults (44%). Similar trends appeared in children (47% vs. 42%). Motor and sensory symptoms were more frequent in FND-only adults and children compared to FND + Autism, but this pattern did not remain significant in children following a Bonferroni-corrected significance threshold (*α* = 0.008).

### Comorbidities

**Fig. 4:**
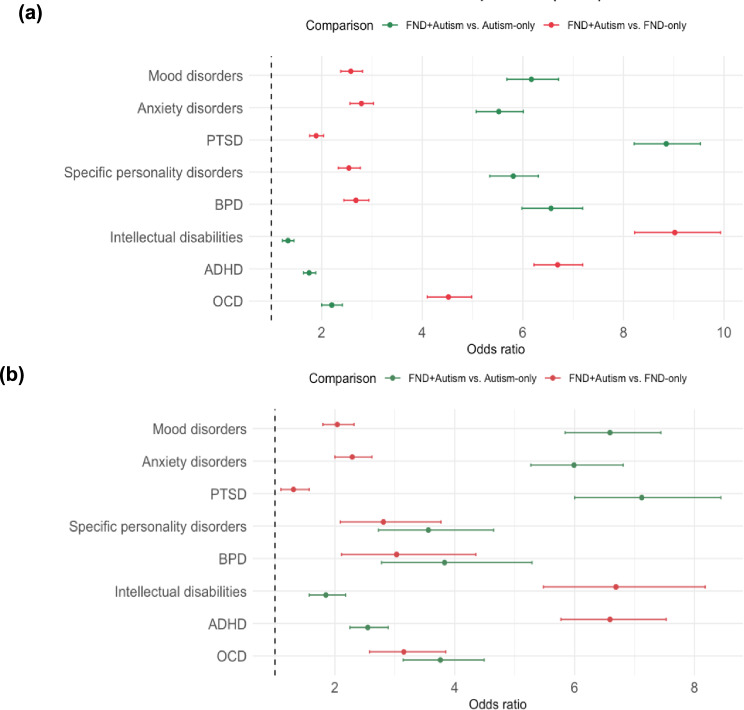
Forest plot of odds ratios for the FND+Autism group compared to FND-only and Autism-only in (**a**) adults and (**b**) children Note. PTSD = Post-Traumatic Stress Disorder; BPD = Borderline Personality Disorder; OCPD = Obsessive-Compulsive Personality Disorder; ADHD = Attention-Deficit Hyperactivity Disorder; OCD = Obsessive-Compulsive Disorder.

Comorbidities for both adults and children are presented in Table 3, and chi-square analysis and effect sizes in Supplementary Table S6, and odds ratios in Fig. 4 and Supplementary Table S7. Table S8 in the supplementary information shows comorbidity rates for all FND patients, as well as the FND-only and FND + Autism subgroups. As the FND-only group is much larger than the FND + Autism group, comorbidity percentages in the total FND sample largely match those in the FND-only group (to within 0–2% in the adult and 0–4% in the child subsamples).

**Table 3:** a Comorbidity rates across diagnostic groups in adult cohort. b. Comorbidity rates across diagnostic groups in child cohort

(a)			
	FND + Autism *n* = 3178	FND-only *n* = 85746	Autism-only *n* = 235245
	*n *(%)	*n *(%)	*n *(%)
Mood disorders	2438 (77%)	48059 (56%)	81862 (35%)
Anxiety disorders	2479 (78%)	48021 (56%)	92006 (39%)
PTSD	1119 (35%)	19124 (22%)	13616 (6%)
Specific personality disorders^a^	748 (24%)	9266 (11%)	11846 (5%)
BPD	589 (19%)	6719 (8%)	7887 (3%)
OCPD	12 (< 1%)	148 (< 1%)	506 (< 1%)
Intellectual disabilities	665 (21%)	2440 (3%)	38945 (17%)
ADHD	1590 (50%)	11167 (13%)	85482 (36%)
OCD	567 (18%)	3386 (4%)	19104 (8%)
	FND + Autism *n* = 1067	FND-only *n* = 11155	Autism-only *n* = 217893
	*n *(%)	*n *(%)	*n *(%)
Mood disorders	537 (50%)	3699 (33%)	29023 (13%)
Anxiety disorders	718 (67%)	5279 (47%)	55706 (26%)
PTSD	158 (15%)	1305 (12%)	5196 (2%)
Specific personality disorders^a^	58 (5%)	224 (2%)	3460 (2%)
BPD	39 (4%)	138 (1%)	2135 (1%)
OCPD	10 (< 1%)	10 (< 1%)	115 (< 1%)
Intellectual disabilities	169 (16%)	305 (3%)	20124 (9%)
ADHD	680 (64%)	2348 (21%)	88805 (41%)
OCD	141 (13%)	468 (4%)	7664 (4%)

*Note.* ^a^Percentages are of the total sample, not of the specific personality disorder category

PTSD = Post-Traumatic Stress Disorder; BPD = Borderline Personality Disorder; OCPD = Obsessive-Compulsive Personality Disorder; ADHD = Attention-Deficit Hyperactivity Disorder; OCD = Obsessive-Compulsive Disorder

Overall, the FND + Autism group showed significantly higher rates of all assessed comorbidities compared to both FND-only and Autism-only groups. All comparisons remained statistically significant at *p* < 0.05 and after Bonferroni correction (adjusted *α* = 0.007). Effect sizes, indicated using the phi coefficient (ϕ), ranged from small to moderate (child cohort: 0.02–0.28; adult cohort: 0.01–0.20).

Mood and anxiety disorders were the most common comorbidities across both adults and children. About three-quarters of adults with FND + Autism had either condition, compared to just over half of FND-only and about one-third of Autism-only (Table 3). Children showed a similar pattern, with around half of the FND + Autism group affected by mood disorder and two-thirds by anxiety. Odds ratios indicated FND + Autism adults and children were twice as likely than FND-only, and up to six times more likely than Autism-only, to receive these diagnoses. Effect sizes were small in adults and children (*ϕ* = 0.08-0.10).

PTSD was more frequent in FND + Autism adults, with a moderate effect size compared to Autism-only (*ϕ* = 0.14). Children showed lower rates but a similar pattern. OCD was also significantly higher in the FND + Autism group, with moderate effects compared to FND-only in both adults (*ϕ* = 0.13) and children (*ϕ* = 0.12). Adults with FND + Autism were over four times more likely to have OCD than the FND-only, and twice as likely as the Autism-only group. Children with FND + Autism had approximately three times higher odds of OCD than both comparison groups.

Personality disorders were elevated in FND + Autism compared to FND-only and Autism-only, with lower rates in children overall. Effect sizes were small for specific personality disorder across both ages (0.02–0.09), with similar effect sizes for BPD. OCPD diagnoses were negligible across all groups for the adult (0.17–0.37%) and child cohorts (0.05–0.94%) and were therefore not included in further analysis.

Both intellectual disabilities and ADHD were significantly more common in FND + Autism across both age cohorts. ADHD was diagnosed in half of adults and two-thirds of children with FND + Autism, with moderate effect sizes versus FND-only (*ϕ* = 0.20 in adults; *ϕ* = 0.28 in children).

Intellectual disabilities were also more common in the FND + Autism group, yielding moderate effect sizes in comparison to FND-only (*ϕ* = 0.18 adults; *ϕ* = 0.19 children), but smaller effect sizes compared to Autism-only (*ϕ* < 0.02).

### Sex stratification

Analyses stratified by sex are presented in Supplementary Tables S9–S12 (see supplementary information). Overall, results were consistent with the main analyses. Most comorbidities and subtype differences remained significant in both sexes, though some findings did not survive Bonferroni correction, likely reflecting reduced sample sizes. For example, BPD and PTSD rates in male children did not differ between FND + Autism and FND-only. Similarly, in adults, Motor and Other subtypes did not reach significance in males, and the Mixed subtype did not reach significance in females.

## Discussion

We present results from the largest study of people to date with co-occurring FND and autism diagnoses, compared those with FND or autism only. People with a dual-diagnosis were more likely to experience functional seizures than those with FND-only, and had higher rates of comorbidities than either FND- or Autism-only groups, most notably ADHD. This data expands knowledge on the overlap between autism and FND and the characteristics of the dual-diagnosed population.

### Evidence for increased rates of autism in FND

Patients with FND + Autism comprised over 2% of the FND population and 0.76% of the autism population in TriNetX. To our knowledge, this is the first estimate of the rate of FND in an autistic population and is higher than the 0.08–0.14% FND prevalence in the general population [1]. The rates of autism were 5.7 times higher in FND compared to the base rates of the TriNetX population.

Over half of the FND + Autism group had autism diagnosed before FND, indicating many individuals navigate the implications of two diagnoses without their neurodevelopmental differences been previously recognised and supported. Similarly, 34% received their autism diagnosis in childhood, highlighting a sizable population with autism first diagnosed in adulthood—consistent with under-recognition of autism, especially in females and those without intellectual disability.

For some, a diagnosis of autism in adulthood can provide explanations and affirming support [22], for others, obtaining two potentially stigmatising diagnoses may be difficult to adapt to [23, 24]. Future research would benefit from exploring this experience through qualitative methodologies, actively incorporating the voices of people with diagnoses of both FND and autism, especially given the historical exclusion of autistic perspectives in research [25].

Notably, the rate of FND and autism observed in this study was lower than that reported in Tamilson et al. [3]. One factor accounting for this is that the estimates may not be directly comparable due to methodological differences. Our estimate was derived from a large-scale global database using clinician-assigned diagnostic codes, while Tamilson et al.’s figure reflects a synthesis of smaller, heterogenous studies, many of which used variable diagnostic criteria and focussed on specific FND subtypes. Both estimates provide valid insights but should be interpreted within the context of an emerging body of research. As interest in the intersection between autism and FND increases and studies employing more diverse methodologies emerge, prevalence estimates will likely become more precise and reflective of the broader clinical population.

Similarly, the background autism prevalence in the TriNetX population of 0.38% is lower than global estimates of 1–2% [4], which we suspected was due to shifts in diagnostic practice in recent years [21]. We therefore performed additional analyses stratified by age. In the background population, autism was recorded in 0.23% of adults and 1.8% of children. Among individuals with FND, autism was present in 2.2% of adults and 9.6% of children. This equated to autism being 5.3 times more common in children with FND and 9.7 times more common in adults with FND, showing that while the ~ sixfold increase held for children, adults with FND were ever more likely to have an autism diagnosis compared to the base rate in the adult population. It is possible that this effect is in part driven by a late diagnosis of autism in adults presenting with FND.

### Presence of FND subtypes

Seizures were the most frequent FND subtype in both adults and children across groups, with FND + Autism having significantly higher percentage of seizures than FND-only. Conversely, motor and sensory symptoms were higher in adult FND-only but did not differ significantly in children. This suggests seizures may be a particularly prominent feature in adults with FND + Autism, and points toward distinct clinical characteristics by age group [18].

A contributing factor towards this may be the high prevalence of epilepsy in autism (~ 12.1%) [26], however this was not looked at in this dataset due to the nature of the database retaining historical diagnoses, and the common misdiagnosis of functional seizures as epileptic seizures [e.g. 27].

Previous work implicates interoceptive difficulties and alexithymia in functional seizures [28], and these are common in autism [7], suggesting a potential link between autism/autistic cognition and the manifestation of seizure symptoms in FND.

### Psychiatric comorbidities

Across all comorbidities, FND + Autism had significantly higher rates than FND-only or Autism-only, consistent across ages, highlighting the vulnerability of the dual-diagnosed group. This is notable as functional seizures are associated with higher psychiatric comorbidities than functional movement disorders [10], suggesting the interplay between autism, functional seizures, and elevated psychiatric comorbidities warrants further investigation.

Anxiety and mood disorders affected 70% of FND + Autism adults, exceeding rates expected from either condition alone [29, 30]. This indicates possible compounding vulnerability in FND + Autism, though interaction effects were not tested.

PTSD was also elevated in FND + Autism, consistent with the role of trauma as a notable—but not sole nor necessary—risk factor for FND [10, 31], and raising interesting questions surrouding the intersection of autism and trauma vulnerability in the context of FND. Autistic individuals face higher trauma exposure, including interpersonal [32, 33] and sensory-related traumas, potentially stemming from conflicts between autistic characteristics and the environment [34]. Autistic adults are four times more likely to have PTSD than non-autistic adults [33], thus the role of traumatic experiences in autistic people’s vulnerability to developing FND requires focused research.

Personality disorders, especially BPD, were more frequent in adults with co-occurring FND and autism, consistent with prior reports of elevated rates of BPD in FND and overlapping symptoms complicating diagnosis in autism, particularly in women [35]. This aligns with broader discussions around the potential overrepresentation of personality disorders in FND, and how misdiagnosis of BPD in autistic people may further complicate this.

OCPD, however, was not common in our dataset, with < 1% across groups. OCPD is increasingly anecdotally noted in FND patients in clinical settings, but there is currently limited literature supporting an elevated presence. One study by Demartini et al. [12] reported a higher prevalence of OCPD in patients with functional motor symptoms, and suggested possible links with alexithymia and FND. However, this relationship remains unclear and requires further study.

OCD was elevated in FND + Autism with moderate effect sizes, with adults with FND + Autism over four times more likely to have OCD than FND-only, and twice as likely as Autism-only adults. While OCD in the context of FND + Autism is not widely discussed in the literature, it is recognised in autism, with 17–37% of young people experiencing OCD symptoms [36, 37]. However, the link between FND and OCD is less well-established, with one study reporting increased obsessive–compulsive symptoms in patients with Functional Movement Disorder, a subtype of FND [38]. These findings may hint at a cumulative burden in individuals with both FND and autism, but the limited data precludes strong conclusions, and further research is needed.

Higher psychiatric comorbidities in FND + Autism may reflect neurobiological predispositions and psychosocial challenges associated with living with each condition [31, 39]. In autism, psychiatric issues may emerge from both intrinsic vulnerability and social-environmental mismatch, exacerbated by delayed diagnosis and lack of support [23]. In FND, comorbidities may contribute to symptom development or arise post-onset [28]. The co-occurrence of autism and FND may therefore create a complex clinical profile in which challenges associated with each condition compound one another.

### Intellectual disabilities

Intellectual disabilities were notably higher in FND + Autism adults; nearly 10 times more likely than FND-only and 1.4 times more than Autism-only. While roughly 1 in 3 autistic children have an intellectual disability [40], the prevalence of intellectual disabilities in FND remains poorly characterised in the literature. Across all FND patients in our cohort, 3–4% had an intellectual disability diagnosis, compared with 1% estimated prevalence in general population samples [41]. One study noted cases of functional seizures with autism and intellectual disability [42], but this area remains largely unexplored. If intellectual disability is more common in FND + Autism, this has important clinical implications for assessment and support.

### ADHD

A key finding was that nearly half of adults and 63% of children with FND + Autism had ADHD, compared to estimates rates in the general population of 7.6% in children, 5.6% in adolescents [43] and 3.1% in adults [44]. Given high autism and ADHD co-occurrence [14], an increase in ADHD was expected; however, FND + Autism had nearly twice the ADHD rate of Autism-only, suggesting an unexpected amplification of ADHD in the presence of co-occurring FND and autism.

Limited literature exists on ADHD prevalence in FND. Recent studies using the Adult ADHD Self-Report Scale found 18% of FND patients [45] and 35% of patients with functional seizures [46] warranted an ADHD assessment. ADHD has not been explicitly linked to FND, but attentional phenomena are relevant; for example, in functional movement disorders attention is disproportionately directed towards ongoing visual feedback from the affected body part, and symptoms can improve or even cease with distraction [47]. This raises the possibility that attentional dysregulation, characteristic of ADHD, may intersect with processes involved in FND, though this remains to be explored empirically.

Increased ADHD rates in co-occurring FND + Autism raise pathophysiological questions and treatment implications, discussed in a recent case report [19]. Could ADHD be a risk factor for the development of FND, e.g. via increased injury risk, given that injuries can precede the development of FND [48]? How effective are FND treatments for patients with ADHD and/or co-occurring autism? How could treatment for ADHD affect symptoms of FND? These important questions warrant future exploration.

### Strengths and limitations

A key strength of this study is that it is the largest cohort analysis of people with co-occurring FND and autism diagnoses to date. The breadth of the dataset allows for robust comparisons across diagnostic groups, and the use of clinically assigned diagnoses strengthens the reliability of diagnostic classification and enhances the clinical relevance and translational value of the findings.

There are limitations inherent to the dataset and broader research context that warrant consideration. Notably, the current estimates of diagnosed autism in FND may not fully reflect the true prevalence of autism. While the study benefits from using clinically confirmed autism diagnoses, there is notable underdiagnoses of autism in adults, particularly females, a group highly represented in FND. Therefore, it is likely that some individuals with co-occurring FND and autism were included in the FND-only group, possibly diluting group differences, and potentially accounting for the differences in our estimate compared to the Tamilson estimate. In contrast, the higher rate of autism in the child cohort may offer a more accurate picture, given better recognition of autism in younger populations [21].

Moreover, the subset of individuals who did receive both an autism and FND diagnosis may represent more clinically complex or visible cases. This follows trends within late-diagnosed autistic people who report a history of misdiagnosis and tend to seek help only when co-occurring psychiatric symptoms emerge [13]. As such, it is possible the FND + Autism group may potentially overrepresent the prevalence of psychiatric comorbidities and ADHD in this co-occurring group, with ‘milder’ or less complex cases of FND + Autism being underdiagnosed.

Additionally, it is worth noting that while all group differences were statistically significant, the strength of the associations varied and most of the effect sizes were small. This suggests that while the findings are robust in terms of significance, the magnitude of group differences may be low, and caution should be exercised when interpreting their practical relevance.

The present study also utilised medical records of clinically diagnosed conditions. While this offers the strength of capturing well-established diagnoses, it is also reliant on historical clinical documentation, which may be incomplete or inconsistent across healthcare providers. In particular, coding FND using ICD-10 codes has proven difficult, with previous studies finding clinicians can be hesitant to use the F44 category for a patient with FND due to concerns that FND diagnosis might be incorrect, preferring to code for symptoms until the diagnostic work-up is definitive, and difficulty selecting the right FND-related diagnostic code [49].

Similarly, as TriNetX captures cumulative diagnoses, some conditions may reflect earlier, subsequently revised diagnoses. For instance, the elevated rates of BPD in autism could partly reflect individuals first diagnosed with BPD before receiving an autism diagnosis [11]. It is also not possible to determine whether the psychiatric diagnoses preceded or followed the onset of FND. Future studies should investigate the temporal relationship to better understand the trajectory of these comorbidities.

Finally, for the purpose of clarity for future meta-analyses or systematic reviews, we have papers in preparation using the TriNetX database addressing different aspects of FND including comorbidities other than autism.

## Conclusion

This study presents findings from a large-scale database of people with co-occurring FND and autism diagnoses, offering an overview and characterisation of those with a dual diagnosis. The results indicate that people with both FND and autism have a distinct clinical profile, characterised by higher rates of functional seizures and psychiatric comorbidities, and an especially high prevalence of ADHD and OCD compared to those with FND or autism alone. These findings highlight the importance of recognising the complexity of co-occurring FND and autism and suggest dual-diagnosed individuals may have unique clinical and support needs. This work provides a platform for future research and underscores the need for neurodiversity-informed diagnostic procedures and treatment approaches in FND.

## Supplementary Information

Below is the link to the electronic supplementary material.

Supplementary file1 (DOCX 83 kb)

## Data Availability

The data used in this study were obtained from the TriNetX research network. While the aggregated data analysed in this study could be made available upon reasonable request, access to the platform requires a license and appropriate permissions.
