# Supplementary material for: Co-occurring functional neurological disorder and autism: an exploratory study of comorbidities in a retrospective cohort study using TriNetX
Source: J Neurol. 2025 Sep 25;272(10):653. doi: 10.1007/s00415-025-13385-6 (PMC12464146; doi:10.1007/s00415-025-13385-6)
Supplement: Supplementary file 1 — Supplementary file1 (DOCX 83 kb) [file 415_2025_13385_MOESM1_ESM.docx]

**The RECORD statement – checklist of items, extended from the STROBE statement, that should be reported in observational studies using routinely collected health data.**

|  | **Item No.** | **STROBE items** | **Location in manuscript where items are reported** | **RECORD items** | **Location in manuscript where items are reported** |
| --- | --- | --- | --- | --- | --- |
| **Title and abstract** | | | | | |
|  | 1 | (a) Indicate the study’s design with a commonly used term in the title or the abstract (b) Provide in the abstract an informative and balanced summary of what was done and what was found | Abstract – methods section | RECORD 1.1: The type of data used should be specified in the title or abstract. When possible, the name of the databases used should be included.  RECORD 1.2: If applicable, the geographic region and timeframe within which the study took place should be reported in the title or abstract.  RECORD 1.3: If linkage between databases was conducted for the study, this should be clearly stated in the title or abstract. | Title  N/A  N/A |
| **Introduction** | | | | | |
| Background rationale | 2 | Explain the scientific background and rationale for the investigation being reported | Background |  |  |
| Objectives | 3 | State specific objectives, including any prespecified hypotheses | Background |  |  |
| **Methods** | | | | | |
| Study Design | 4 | Present key elements of study design early in the paper | Background + Method - *TriNetX Research Network, Inclusion of comorbidities, Statistical Analysis* |  |  |
| Setting | 5 | Describe the setting, locations, and relevant dates, including periods of recruitment, exposure, follow-up, and data collection | Method – *TriNetX Research Network* |  |  |
| Participants | 6 | *(a)* ***Cohort study* - Give the eligibility criteria, and the sources and methods of selection of participants. Describe methods of follow-up**  *Case-control study* - Give the eligibility criteria, and the sources and methods of case ascertainment and control selection. Give the rationale for the choice of cases and controls  *Cross-sectional study* - Give the eligibility criteria, and the sources and methods of selection of participants  *(b)* ***Cohort study* - For matched studies, give matching criteria and number of exposed and unexposed**  *Case-control study* - For matched studies, give matching criteria and the number of controls per case | Method – *Study population*  N/A | RECORD 6.1: The methods of study population selection (such as codes or algorithms used to identify subjects) should be listed in detail. If this is not possible, an explanation should be provided.  RECORD 6.2: Any validation studies of the codes or algorithms used to select the population should be referenced. If validation was conducted for this study and not published elsewhere, detailed methods and results should be provided.  RECORD 6.3: If the study involved linkage of databases, consider use of a flow diagram or other graphical display to demonstrate the data linkage process, including the number of individuals with linked data at each stage. | Method – *Study population + Table 1*  Used ICD-10 diagnostic codes for autism + asperger (autism spectrum) and FND agreed upon by clinicians  N/A |
| Variables | 7 | Clearly define all outcomes, exposures, predictors, potential confounders, and effect modifiers. Give diagnostic criteria, if applicable. | Background + Methods – *Inclusion of comorbidities + Table A1* | RECORD 7.1: A complete list of codes and algorithms used to classify exposures, outcomes, confounders, and effect modifiers should be provided. If these cannot be reported, an explanation should be provided. | Methods – *Inclusion of comorbidities + Table A1* |
| Data sources/ measurement | 8 | For each variable of interest, give sources of data and details of methods of assessment (measurement).  Describe comparability of assessment methods if there is more than one group | Methods – *Inclusion of comorbidities + Table A1*  Diagnoses identified using ICD-10 codes as used by TriNetX |  |  |
| Bias | 9 | Describe any efforts to address potential sources of bias | Method – *Study population*  Sex differences in autism and FND population discussed and the same analyses used in the main results were run for sex stratified and reported in appendix 3 |  |  |
| Study size | 10 | Explain how the study size was arrived at | Method – *Study population*  Study size was determined by identifying all individuals meeting diagnostic criteria within TriNetX database at the time of data extraction in line with the age truncated groups. No a priori sample size calculation was performed, as the study used available electronic health records to include all eligible cases. |  |  |
| Quantitative variables | 11 | Explain how quantitative variables were handled in the analyses. If applicable, describe which groupings were chosen, and why | Method – *Inclusion of comorbidities; Statistical analysis*  Quantitative variables were categorized into clinically meaningful groups based on diagnostic codes (e.g., age groups: children vs. adults) to facilitate comparison across cohorts. Statistical analyses primarily used categorical groupings (e.g., presence or absence of comorbidities) assessed via chi-square tests to evaluate differences between groups. |  |  |
| Statistical methods | 12 | (a) Describe all statistical methods, including those used to control for confounding  (b) Describe any methods used to examine subgroups and interactions  (c) Explain how missing data were addressed  (d) *Cohort study* - If applicable, explain how loss to follow-up was addressed  *Case-control study* - If applicable, explain how matching of cases and controls was addressed  *Cross-sectional study* - If applicable, describe analytical methods taking account of sampling strategy  (e) Describe any sensitivity analyses | (a) Methods – *Statistical analysis*  (b) Sex-stratified analyses are described in the Methods – Statistical Analysis and Appendix 3  (c) N/A  (d) Not matched; stratified to account for group demographic differences  (e) Not conducted |  |  |
| Data access and cleaning methods |  | .. |  | RECORD 12.1: Authors should describe the extent to which the investigators had access to the database population used to create the study population.  RECORD 12.2: Authors should provide information on the data cleaning methods used in the study. | The investigators accessed de-identified, aggregated data via the TriNetX platform; direct individual-level data were not available. (See Methods – *TriNetX Research Network*  No additional data cleaning was performed as the dataset consisted of de-identified, clinically coded records with no missing key variables relevant to the analyses. |
| Linkage |  | .. |  | RECORD 12.3: State whether the study included person-level, institutional-level, or other data linkage across two or more databases. The methods of linkage and methods of linkage quality evaluation should be provided. | This study did not involve linkage across two or more databases; analyses were conducted using a single de-identified electronic health records database (TriNetX). |
| **Results** | | | | | |
| Participants | 13 | (a) Report the numbers of individuals at each stage of the study (*e.g.*, numbers potentially eligible, examined for eligibility, confirmed eligible, included in the study, completing follow-up, and analysed)  (b) Give reasons for non-participation at each stage.  (c) Consider use of a flow diagram | **(a) and (b):** Numbers of individuals at each stage and reasons for truncation are reported in the Methods - *Study Population* section and reported in Results – *FND and Autism Diagnosis*  **(c):** A flow diagram illustrating participant selection is included in Appendix Figure A1 and mentioned in text Methods – *Study population* | RECORD 13.1: Describe in detail the selection of the persons included in the study (*i.e.,* study population selection) including filtering based on data quality, data availability and linkage. The selection of included persons can be described in the text and/or by means of the study flow diagram. | Methods - *Study Population* |
| Descriptive data | 14 | (a) Give characteristics of study participants (*e.g.*, demographic, clinical, social) and information on exposures and potential confounders  (b) Indicate the number of participants with missing data for each variable of interest  (c) *Cohort study* - summarise follow-up time (*e.g.*, average and total amount) | (a) Methods - *Study Population, Table 2*  (b) N/A no missing data |  |  |
| Outcome data | 15 | *Cohort study* - Report numbers of outcome events or summary measures over time  *Case-control study* - Report numbers in each exposure category, or summary measures of exposure  *Cross-sectional study* - Report numbers of outcome events or summary measures | Outcome events are reported as rates within groups, with no longitudinal incidence data available. |  |  |
| Main results | 16 | (a) Give unadjusted estimates and, if applicable, confounder-adjusted estimates and their precision (e.g., 95% confidence interval). Make clear which confounders were adjusted for and why they were included  (b) Report category boundaries when continuous variables were categorized  (c) If relevant, consider translating estimates of relative risk into absolute risk for a meaningful time period | (a) Unadjusted estimates (chi-square group comparisons) are reported; no confounder-adjusted estimates were performed as this study was descriptive.  (b) Continuous variables (e.g. age) were categorised into child and adult groups based on clinical relevance  (c) N/A as study does not report relative risks or longitudinal outcomes |  |  |
| Other analyses | 17 | Report other analyses done—e.g., analyses of subgroups and interactions, and sensitivity analyses | Odds ratios, phi coefficient effect sizes |  |  |
| **Discussion** | | | | | |
| Key results | 18 | Summarise key results with reference to study objectives | Discussion (all) |  |  |
| Limitations | 19 | Discuss limitations of the study, taking into account sources of potential bias or imprecision. Discuss both direction and magnitude of any potential bias | Discussion – *Strengths and limitations* | RECORD 19.1: Discuss the implications of using data that were not created or collected to answer the specific research question(s). Include discussion of misclassification bias, unmeasured confounding, missing data, and changing eligibility over time, as they pertain to the study being reported. | Discussion – *Strengths and limitations* |
| Interpretation | 20 | Give a cautious overall interpretation of results considering objectives, limitations, multiplicity of analyses, results from similar studies, and other relevant evidence | Discussion (all) |  |  |
| Generalisability | 21 | Discuss the generalisability (external validity) of the study results | Discussion (all) |  |  |
| **Other Information** | | | | | |
| Funding | 22 | Give the source of funding and the role of the funders for the present study and, if applicable, for the original study on which the present article is based | N/A |  |  |
| Accessibility of protocol, raw data, and programming code |  | .. |  | RECORD 22.1: Authors should provide information on how to access any supplemental information such as the study protocol, raw data, or programming code. |  |

*Reference: Benchimol EI, Smeeth L, Guttmann A, Harron K, Moher D, Petersen I, Sørensen HT, von Elm E, Langan SM, the RECORD Working Committee. The REporting of studies Conducted using Observational Routinely-collected health Data (RECORD) Statement. *PLoS Medicine* 2015; in press.

*Checklist is protected under Creative Commons Attribution ([CC BY](http://creativecommons.org/licenses/by/4.0/)) license.

**SUPPLEMENTARY MATERIAL**

| **Table 2. ICD-10-CM Codes Included in the Analysis** | | |
| --- | --- | --- |
| **F30 - F39: Mood [affective] disorders** | F30 – Manic episode | |
|  | F31 - Bipolar disorder | |
|  | F32 - Depressive episode | |
|  | F33 - Major depressive disorder, recurrent | |
|  | F34 – Persistent mood [affective] disorder | |
| F40-F48: Anxiety, dissociative, stress-related, somatoform | **F41 - Other Anxiety Disorders** | |
|  |  | F41.0 - Panic Disorder |
|  |  | F41.1 - Generalised Anxiety Disorder |
|  | **F42 – Obsessive-Compulsive Disorder** | |
|  | F43 - Reaction to severe stress, and adjustment disorder | |
|  |  | **F43.1 - Post-traumatic stress disorder** |
|  |  |  |
| F60 - F69: Disorders of adult personality and behaviour | **F60 - Specific personality disorders** | |
|  |  | **F60.3 - Borderline Personality Disorder** |
|  |  | **F60.5 – Obsessive-Compulsive Personality Disorder** |
| **F70 - F79: Intellectual Disabilities** |  | |
| F90 - F98: Behavioural and emotional disorders with onset usually occurring in childhood and adolescence | **F90 – ADHD** | |

| **Table 4a. Chi-Square Analysis of FND subtypes in Adults** | | | | | | | | |
| --- | --- | --- | --- | --- | --- | --- | --- | --- |
|  |  |  | | |  |  | | |
|  |  | FND+Autism  *n* = 3178 | | FND-only  *n* = 85746 |  | Chi-Square | | |
|  |  | *n*(%) | | *n*(%) |  | *χ²* | *p* | φ |
| Motor |  | 929 (29%) | | 28175 (33%) |  | 18.14 | <.001** | 0.01 |
| Seizures |  | 1654 (52%) | | 37434 (44%) |  | 87.19 | <.001** | 0.03 |
| Sensory |  | 554 (17%) | | 19622 (23%) |  | 51.61 | <.001** | 0.02 |
| Mixed |  | 226 (7%) | | 5317 (6%) |  | 4.19 | .04* | 0.01 |
| Other |  | 447 (14%) | | 8657 (10%) |  | 52.11 | <.001** | 0.02 |
| Unspecified |  | 839 (26%) | | 21933 (26%) |  | 1.01 | .31 | <0.01 |
| *Note.* All Chi-square tests degrees of freedom = 1; *χ²* = chi-square statistic; *p* = significance level; * = significant at *p* <.05; ** = significant at *p* <.01; φ = phi (effect size). | | | | | | | | |
| **Table 4b. Chi-Square Analysis of FND subtypes in Children** | | | | | | | | |
|  |  | FND+Autism  *n* = 1067 | FND-only  *n* = 11155 | |  | Chi-Square | | |
|  |  | *n*(%) | | *n*(%) |  | *χ²* (df) | *p* | φ |
| Motor |  | 343 (32%) | | 4042 (36%) |  | 6.90 | .009^†^ | 0.02 |
| Seizures |  | 504 (47%) | | 4682 (42%) |  | 10.83 | <.001** | 0.03 |
| Sensory |  | 179 (17%) | | 1734 (16%) |  | 1.03 | .31 | 0.01 |
| Mixed |  | 139 (13%) | | 1503 (13%) |  | 0.13 | .72 | <0.01 |
| Other |  | 80 (7%) | | 647 (6%) |  | 4.72 | .03* | 0.02 |
| Unspecified |  | 199 (19%) | | 2152 (19%) |  | 0.22 | .64 | <0.01 |
| *Note.*  All Chi-square tests degrees of freedom = 1; *χ²* = chi-square statistic; *p* = significance level; * = significant at *p* <.05; ** = significant at *p* <.01; ^†^ = not significant after Bonferroni correction; φ = phi (effect size). | | | | | | | | |

|  | **Table 6a. Chi-Square Analysis of Comorbidities in Adult Cohort** | | | | | | | | | | |
| --- | --- | --- | --- | --- | --- | --- | --- | --- | --- | --- | --- |
|  | |  | FND+Autism vs. FND-only | | |  | FND+Autism vs. Autism-only | | |  |  |
|  | |  | *χ²* | *p* | φ |  | *χ²* | *p* | φ |  |  |
| Mood disorders | |  | 532.53 | <.001** | 0.08 |  | 2408.60 | <.001** | 0.10 |  |  |
| Anxiety disorders | |  | 603.59 | <.001** | 0.08 |  | 1981.10 | <.001** | 0.09 |  |  |
| PTSD | |  | 289.65 | <.001** | 0.06 |  | 4676.60 | <.001** | 0.14 |  |  |
| Specific personality disorders | |  | 495.70 | <.001** | 0.07 |  | 2141.60 | <.001** | 0.09 |  |  |
| BPD | |  | 463.52 | <.001** | 0.07 |  | 2103.30 | <.001** | 0.09 |  |  |
| Intellectual disabilities | |  | 2967.10 | <.001** | 0.18 |  | 42.91 | <.001** | 0.01 |  |  |
| ADHD | |  | 3412.60 | <.001** | 0.20 |  | 253.05 | <.001** | 0.03 |  |  |
| OCD | |  | 1389.1 | <.001** | 0.13 |  | 390.12 | <.001** | 0.04 |  |  |
| *Note.* All Chi-square tests degrees of freedom = 1; *χ²* = chi-square statistic; *df* = degrees of freedom *p* = significance level; * = significant at *p* <.05; ** = significant at *p* <.01; φ = phi (effect size). All comparisons significant after Bonferroni adjusted significance threshold (α = 0.006).  PTSD = Post-Traumatic Stress Disorder; BPD = Borderline Personality Disorder; ADHD = Attention-Deficit Hyperactivity Disorder; OCD = Obsessive-Compulsive Disorder. | | | | | | | | | | |  |
|  | **Table 6b. Chi-Square Analysis of Comorbidities in Child Cohort** | | | | | | | | | |  |
|  | |  | FND+Autism vs. FND-only | | |  | FND+Autism vs. Autism-only | | |  |  |
|  | |  | *χ²* | *p* | φ |  | *χ²* | *p* | φ |  |  |
| Mood disorders | |  | 125.99 | <.001** | 0.10 |  | 1242.20 | <.001** | 0.08 |  |  |
| Anxiety disorders | |  | 154.56 | <.001** | 0.11 |  | 964.24 | <.001** | 0.07 |  |  |
| PTSD | |  | 8.64 | 0.003 | 0.03 |  | 681.79 | <.001** | 0.06 |  |  |
| Specific personality disorders | |  | 49.25 | <.001** | 0.06 |  | 97.03 | <.001** | 0.02 |  |  |
| BPD | |  | 38.22 | <.001** | 0.06 |  | 74.61 | <.001** | 0.02 |  |  |
| Intellectual disabilities | |  | 445.11 | <.001** | 0.19 |  | 54.27 | <.001** | 0.02 |  |  |
| ADHD | |  | 949.61 | <.001** | 0.28 |  | 230.95 | <.001** | 0.03 |  |  |
| OCD | |  | 165.42 | <.001** | 0.12 |  | 287.65 | <.001** | 0.07 |  |  |
| *Note.* All Chi-square tests degrees of freedom = 1; *χ²* = chi-square statistic; *df* = degrees of freedom *p* = significance level; * = significant at *p* <.05; ** = significant at *p* <.01; φ = phi (effect size). All comparisons significant after Bonferroni adjusted significance threshold (α = 0.006).  PTSD = Post-Traumatic Stress Disorder; BPD = Borderline Personality Disorder; ADHD = Attention-Deficit Hyperactivity Disorder; OCD = Obsessive-Compulsive Disorder. | | | | | | | | | | |  |

|  | **Table 7a. Odds ratios for Comorbidities in the Adult Cohort** | | | | | | | | | | |
| --- | --- | --- | --- | --- | --- | --- | --- | --- | --- | --- | --- |
|  | |  | FND+Autism vs. FND-only | | |  | FND+Autism vs. Autism-only | | |  |  |
|  | |  | OR | 95% CI Lower | 95% CI Upper |  | OR | 95% CI Lower | 95% CI Upper |  |  |
| Mood disorders | |  | 2.58 | 2.38 | 2.81 |  | 6.17 | 5.68 | 6.71 |  |  |
| Anxiety disorders | |  | 2.79 | 2.56 | 3.03 |  | 5.52 | 5.07 | 6.01 |  |  |
| PTSD | |  | 1.89 | 1.76 | 2.04 |  | 8.85 | 8.21 | 9.53 |  |  |
| Specific personality disorders | |  | 2.54 | 2.33 | 2.77 |  | 5.81 | 5.34 | 6.31 |  |  |
| BPD | |  | 2.68 | 2.44 | 2.94 |  | 6.56 | 5.98 | 7.19 |  |  |
| Intellectual disabilities | |  | 9.02 | 8.22 | 9.93 |  | 1.33 | 1.22 | 1.45 |  |  |
| ADHD | |  | 6.69 | 6.22 | 7.19 |  | 1.75 | 1.64 | 1.88 |  |  |
| OCD | |  | 4.52 | 4.10 | 4.98 |  | 2.20 | 2.00 | 2.41 |  |  |
| *Note.* OR = Odds ratio; CI = Confidence Interval; PTSD = Post-Traumatic Stress Disorder; BPD = Borderline Personality Disorder; ADHD = Attention-Deficit Hyperactivity Disorder; OCD = Obsessive-Compulsive Disorder. | | | | | | | | | | |  |
|  | **Table 7b. Odds ratios for Comorbidities in the Child Cohort** | | | | | | | | | |  |
|  | |  | FND+Autism vs. FND-only | | |  | FND+Autism vs. Autism-only | | |  |  |
|  | |  | OR | 95% CI Lower | 95% CI Upper |  | OR | 95% CI Lower | 95% CI Upper |  |  |
| Mood disorders | |  | 2.04 | 1.80 | 2.32 |  | 6.59 | 5.84 | 7.44 |  |  |
| Anxiety disorders | |  | 2.29 | 2.00 | 2.62 |  | 5.99 | 5.27 | 6.81 |  |  |
| PTSD | |  | 1.31 | 1.10 | 1.57 |  | 7.12 | 6.00 | 8.44 |  |  |
| Specific personality disorders | |  | 2.81 | 2.09 | 3.77 |  | 3.56 | 2.73 | 4.65 |  |  |
| BPD | |  | 3.03 | 2.11 | 4.35 |  | 3.83 | 2.78 | 5.29 |  |  |
| Intellectual disabilities | |  | 6.69 | 5.48 | 8.18 |  | 1.85 | 1.57 | 2.18 |  |  |
| ADHD | |  | 6.59 | 5.77 | 7.53 |  | 2.55 | 2.25 | 2.89 |  |  |
| OCD | |  | 3.15 | 2.58 | 3.85 |  | 3.76 | 3.14 | 4.49 |  |  |
| *Note.* OR = Odds ratio; CI = Confidence Interval; PTSD = Post-Traumatic Stress Disorder; BPD = Borderline Personality Disorder; ADHD = Attention-Deficit Hyperactivity Disorder; OCD = Obsessive-Compulsive Disorder. | | | | | | | | | | |  |

| **Table 8a. Comorbidity Rates for FND groups in Adult Cohort** | | | | | | | |
| --- | --- | --- | --- | --- | --- | --- | --- |
|  |  | FND - Total  *n* = 88924 |  | FND-only  *n* = 85746 |  | FND+Autism  *n* = 3178 |  |
|  |  | *n (*%) |  | *n (*%) |  | *n (*%) |  |
| Mood disorders |  | 50497 (57%) |  | 48059 (56%) |  | 2438 (77%) |  |
| Anxiety disorders |  | 50500 (57%) |  | 48021 (56%) |  | 2479 (78%) |  |
| PTSD |  | 20243 (23%) |  | 19124 (22%) |  | 1119 (35%) |  |
| Specific personality disorders^1^ |  | 10014 (11%) |  | 9266 (11%) |  | 748 (24%) |  |
| BPD |  | 7308 (8%) |  | 6719 (8%) |  | 589 (19%) |  |
| OCPD |  | 160 (<1%) |  | 12 (<1%) |  | 148 (<1%) |  |
| Intellectual disabilities |  | 3105 (3%) |  | 2440 (3%) |  | 665 (21%) |  |
| ADHD |  | 12757 (14%) |  | 11167 (13%) |  | 1590 (50%) |  |
| OCD |  | 3953 (4%) |  | 3386 (4%) |  | 567 (18%) |  |
| *Note.* ¹Percentages are of the total sample, not of the specific personality disorder category; PTSD = Post-Traumatic Stress Disorder; BPD = Borderline Personality Disorder; OCPD = Obsessive-Compulsive Personality Disorder; ADHD = Attention-Deficit Hyperactivity Disorder; OCD = Obsessive-Compulsive Disorder. | | | | | | | |
| **Table 8b. Comorbidity Rates for FND groups in Child Cohort** | | | | | | | |
|  |  | FND – Total  *n* = 12222 |  | FND-only  *n* = 11155 |  | FND+Autism  *n* = 1067 |  |
|  |  | *n (*%) |  | *n (*%) |  | *n (*%) |  |
| Mood disorders |  | 4236 (35%) |  | 3699 (33%) |  | 537 (50%) |  |
| Anxiety disorders |  | 5997 (49%) |  | 5279 (47%) |  | 718 (67%) |  |
| PTSD |  | 1463 (12%) |  | 1305 (12%) |  | 158 (15%) |  |
| Specific personality disorders^1^ |  | 282 (2%) |  | 224 (2%) |  | 58 (5%) |  |
| BPD |  | 177 (1%) |  | 138 (1%) |  | 39 (4%) |  |
| OCPD |  | 20 (1%) |  | 10 (1%) |  | 10 (0%) |  |
| Intellectual disabilities |  | 474 (4%) |  | 305 (3%) |  | 169 (16%) |  |
| ADHD |  | 3028 (25%) |  | 2348 (21%) |  | 680 (64%) |  |
| OCD |  | 609 (5%) |  | 468 (4%) |  | 141 (13%) |  |
| *Note.* ¹Percentages are of the total sample, not of the specific personality disorder category;  PTSD = Post-Traumatic Stress Disorder; BPD = Borderline Personality Disorder; OCPD = Obsessive-Compulsive Personality Disorder; ADHD = Attention-Deficit Hyperactivity Disorder; OCD = Obsessive-Compulsive Disorder. | | | | | | | |

| **Table 9a. Sex-stratified Chi-Square Analysis of FND subtypes in Adults** | | | | | | | | | | | | | | |
| --- | --- | --- | --- | --- | --- | --- | --- | --- | --- | --- | --- | --- | --- | --- |
|  |  |  | | | | | |  |  | | | | | |
|  |  | **Female** | | | | | |  | **Male** | | | | | |
|  |  | Group Percentage | |  | Chi-Square | | |  | Group Percentage | |  | Chi-Square | | |
|  |  | FND  +Autism | FND-only |  | *χ²* | *p* | φ |  | FND  +Autism | FND-only |  | *χ²* | *p* | φ |
| Motor |  | 28% | 33% |  | 21.02 | <.01** | 0.02 |  | 30% | 33% |  | 4.24 | 0.04^†^ | 0.01 |
| Seizures |  | 55% | 45% |  | 66.08 | <.01** | 0.03 |  | 48% | 39% |  | 42.21 | <.01** | 0.04 |
| Sensory |  | 16% | 23% |  | 45.89 | <.01** | 0.03 |  | 18% | 23% |  | 18.85 | <.01** | 0.03 |
| Mixed |  | 8% | 7% |  | 5.49 | 0.02^†^ | <.01 |  | 6% | 6% |  | 0.54 | 0.46 | <.01 |
| Other |  | 14% | 9% |  | 40.04 | <.01** | 0.02 |  | 15% | 12% |  | 6.56 | 0.01^†^ | 0.02 |
| Unspecified |  | 28% | 26% |  | 1.99 | 0.16 | <.01 |  | 25% | 23% |  | 1.49 | 0.22 | <.01 |
| *Note.* All Chi-square tests degrees of freedom = 1; *χ²* = chi-square statistic; *p* = significance level; * = significant at *p* <.05; ** = significant at *p* <.01; ^†^ = not significant after Bonferroni correction (α = 0.0083); φ = phi (effect size). | | | | | | | | | | | | | | |

| **Table 9b. Sex-stratified Chi-Square Analysis of FND subtypes in Children** | | | | | | | | | | | | | | |
| --- | --- | --- | --- | --- | --- | --- | --- | --- | --- | --- | --- | --- | --- | --- |
|  |  |  | | | | | |  |  | | | | | |
|  |  | **Female** | | | | | |  | **Male** | | | | | |
|  |  | Group Percentage | |  | Chi-Square | | |  | Group Percentage | |  | Chi-Square | | |
|  |  | FND  +Autism | FND-only |  | *χ²* | *p* | φ |  | FND  +Autism | FND-only |  | *χ²* | *p* | φ |
| Motor |  | 36% | 36% |  | 0.08 | 0.78 | <.01 |  | 28% | 36% |  | 10.09 | <.01** | 0.05 |
| Seizures |  | 47% | 43% |  | 2.70 | 0.10 | 0.01 |  | 48% | 39% |  | 14.41 | <.01** | 0.06 |
| Sensory |  | 15% | 15% |  | <.01 | 1.00 | <.01 |  | 18% | 16% |  | 0.93 | 0.34 | 0.02 |
| Mixed |  | 17% | 15% |  | 1.17 | 0.28 | 0.01 |  | 9% | 10% |  | 0.29 | 0.59 | <.01 |
| Other |  | 9% | 5% |  | 12.93 | <.01** | 0.03 |  | 6% | 7% |  | 0.52 | 0.47 | 0.01 |
| Unspecified |  | 21% | 20% |  | 0.28 | 0.59 | <.01 |  | 16% | 17% |  | 0.59 | 0.44 | 0.01 |
| *Note.* All Chi-square tests degrees of freedom = 1; *χ²* = chi-square statistic; *p* = significance level; * = significant at *p* <.05; ** = significant at *p* <.01; ^†^ = not significant after Bonferroni correction; φ = phi (effect size). | | | | | | | | | | | | | | |

| **Table 10a. Comorbidity Rates Across Diagnostic Groups in Adult Cohort - Female** | | | | | | | |
| --- | --- | --- | --- | --- | --- | --- | --- |
|  |  | FND+Autism  *n =* 1835 |  | FND-only  *n =* 63245 |  | Autism-only  *n =* 61490 |  |
|  |  | *n (*%) |  | *n (*%) |  | *n (*%) |  |
| Mood disorders |  | 1501 (82%) |  | 37259 (59%) |  | 27259 (45%) |  |
| Anxiety disorders |  | 1513 (82%) |  | 37398 (59%) |  | 30643 (50%) |  |
| PTSD |  | 792 (43%) |  | 15611 (25%) |  | 6621 (11%) |  |
| Specific personality disorders^1^ |  | 515 (28%) |  | 7404 (12%) |  | 4527 (7%) |  |
| BPD |  | 426 (23%) |  | 5775 (9%) |  | 3451 (6%) |  |
| \| OCPD \|  \| 12 (0%) \|  \| 148 (0%) \|  \| 506 (0%) \| \| --- \| --- \| --- \| --- \| --- \| --- \| --- \| |  | <10 |  | 115 (0%) |  | 153 (0%) |  |
| Intellectual disabilities |  | 353 (19%) |  | 1606 (3%) |  | 10788 (18%) |  |
| ADHD |  | 887 (48%) |  | 7805 (12%) |  | 20475 (33%) |  |
| OCD |  | 335 (18%) |  | 2619 (4%) |  | 5560 (9%) |  |
| *Note.* ¹Percentages are of the total sample, not of the specific personality disorder category; ^2^Counts less than 10 are suppressed by TriNetX for privacy reasons, corresponding percentages are not reported.  PTSD = Post-Traumatic Stress Disorder; BPD = Borderline Personality Disorder; OCPD = Obsessive-Compulsive Personality Disorder; ADHD = Attention-Deficit Hyperactivity Disorder; OCD = Obsessive-Compulsive Disorder. | | | | | | | |
| **Table 10b. Comorbidity Rates Across Diagnostic Groups in Adult Cohort - Male** | | | | | | | |
|  |  | FND+Autism  *n =* 1257 |  | FND-only  *n =* 24801 |  | Autism-only  *n =* 169743 |  |
|  |  | *n (*%) |  | *n (*%) |  | *n (*%) |  |
| Mood disorders |  | 872 (69%) |  | 11783 (69%) |  | 52574 (31%) |  |
| Anxiety disorders |  | 901 (72%) |  | 11470 (46%) |  | 59430 (35%) |  |
| PTSD |  | 284 (23%) |  | 3896 (16%) |  | 6645 (4%) |  |
| Specific personality disorders^1^ |  | 212 (17%) |  | 1860 (7%) |  | 7150 (4%) |  |
| BPD |  | 143 (11%) |  | 930 (4%) |  | 4317 (3%) |  |
| \| OCPD \|  \| 12 (0%) \|  \| 148 (0%) \|  \| 506 (0%) \| \| --- \| --- \| --- \| --- \| --- \| --- \| --- \| |  | <10 |  | 34 (0%) |  | 348 (0%) |  |
| Intellectual disabilities |  | 284 (23%) |  | 870 (4%) |  | 27386 (16%) |  |
| ADHD |  | 661 (53%) |  | 3565 (14%) |  | 63561 (37%) |  |
| OCD |  | 218 (17%) |  | 768 (3%) |  | 13058 (8%) |  |
| *Note.* ¹Percentages are of the total sample, not of the specific personality disorder category; ^2^Counts less than 10 are suppressed by TriNetX for privacy reasons, corresponding percentages are not reported.  BPD = Borderline Personality Disorder; OCPD = Obsessive-Compulsive Personality Disorder; ADHD = Attention-Deficit Hyperactivity Disorder; OCD = Obsessive-Compulsive Disorder | | | | | | | |

|  | **Table 11a. Chi-Square Analysis of Comorbidities in Adult Cohort - Female** | | | | | | | | | | |
| --- | --- | --- | --- | --- | --- | --- | --- | --- | --- | --- | --- |
|  | |  | FND+Autism vs. FND-only | | |  | FND+Autism vs. Autism-only | | |  |  |
|  | |  | *χ²* | *p* | φ |  | *χ²* | *p* | φ |  |  |
| Mood disorders | |  | 386.83 | <.001** | 0.08 |  | 971.42 | <.001** | 0.12 |  |  |
| Anxiety disorders | |  | 402.42 | <.001** | 0.08 |  | 757.19 | <.001** | 0.11 |  |  |
| PTSD | |  | 237.48 | <.001** | 0.06 |  | 1660.00 | <.001** | 0.17 |  |  |
| Specific personality disorders | |  | 443.20 | <.001** | 0.08 |  | 740.19 | <.001** | 0.11 |  |  |
| BPD | |  | 432.51 | <.001** | 0.08 |  | 1010.00 | <.001** | 0.13 |  |  |
| Intellectual disabilities | |  | 2310.70 | <.001** | 0.20 |  | 30.08 | <.001** | 0.02 |  |  |
| ADHD | |  | 1472.00 | <.001** | 0.16 |  | 330.98 | <.001** | 0.07 |  |  |
| OCD | |  | 816.71 | <.001** | 0.11 |  | 178.09 | <.001** | 0.05 |  |  |
| *Note.* All Chi-square tests degrees of freedom = 1; *χ²* = chi-square statistic; *df* = degrees of freedom *p* = significance level; * = significant at *p* <.05; ** = significant at *p* <.01; φ = phi (effect size). All comparisons significant after Bonferroni adjusted significance threshold (α = 0.006). PTSD = Post-Traumatic Stress Disorder; BPD = Borderline Personality Disorder; ADHD = Attention-Deficit Hyperactivity Disorder; OCD = Obsessive-Compulsive Disorder. | | | | | | | | | | |  |
|  | **Table 11b. Chi-Square Analysis of Comorbidities in Adult Cohort - Male** | | | | | | | | | |  |
|  | |  | FND+Autism vs. FND-only | | |  | FND+Autism vs. Autism-only | | |  |  |
|  | |  | *χ²* | *p* | φ |  | *χ²* | *p* | φ |  |  |
| Mood disorders | |  | 228.02 | <.001** | 0.09 |  | 854.57 | <.001** | 0.07 |  |  |
| Anxiety disorders | |  | 309.25 | <.001** | 0.11 |  | 733.08 | <.001** | 0.07 |  |  |
| PTSD | |  | 41.59 | <.001** | 0.04 |  | 1114.9 | <.001** | 0.08 |  |  |
| Specific personality disorders | |  | 142.10 | <.001** | 0.07 |  | 481.83 | <.001** | 0.14 |  |  |
| BPD | |  | 174.32 | <.001** | 0.08 |  | 379.79 | <.001** | 0.05 |  |  |
| Intellectual disabilities | |  | 1025.10 | <.001** | 0.20 |  | 37.91 | <.001** | 0.01 |  |  |
| ADHD | |  | 1282.80 | <.001** | 0.22 |  | 121.31 | <.001** | 0.03 |  |  |
| OCD | |  | 663.02 | <.001** | 0.16 |  | 160.92 | <.001** | 0.03 |  |  |
| *Note.* All Chi-square tests degrees of freedom = 1; *χ²* = chi-square statistic; *df* = degrees of freedom *p* = significance level; * = significant at *p* <.05; ** = significant at *p* <.01; φ = phi (effect size). All comparisons significant after Bonferroni adjusted significance threshold (α = 0.006). PTSD = Post-Traumatic Stress Disorder; BPD = Borderline Personality Disorder; ADHD = Attention-Deficit Hyperactivity Disorder; OCD = Obsessive-Compulsive Disorder. | | | | | | | | | | |  |

|  | **Table 11c. Chi-Square Analysis of Comorbidities in Child Cohort - Female** | | | | | | | | | | |
| --- | --- | --- | --- | --- | --- | --- | --- | --- | --- | --- | --- |
|  | |  | FND+Autism vs. FND-only | | |  | FND+Autism vs. Autism-only | | |  |  |
|  | |  | *χ²* | *p* | φ |  | *χ²* | *p* | φ |  |  |
| Mood disorders | |  | 108.48 | <.001** | 0.11 |  | 605.47 | <.001** | 0.11 |  |  |
| Anxiety disorders | |  | 124.93 | <.001** | 0.12 |  | 483.10 | <.001** | 0.09 |  |  |
| PTSD | |  | 23.86 | <.001** | 0.05 |  | 441.85 | <.001** | 0.09 |  |  |
| Specific personality disorders | |  | 55.97 | <.001** | 0.08 |  | 104.61 | <.001** | 0.05 |  |  |
| BPD | |  | 50.13 | <.001** | 0.08 |  | 106.60 | <.001** | 0.04 |  |  |
| Intellectual disabilities | |  | 406.41 | <.001** | 0.24 |  | 25.59 | <.001** | 0.02 |  |  |
| ADHD | |  | 279.29 | <.001** | 0.17 |  | 90.66 | <.001** | 0.04 |  |  |
| OCD | |  | 142.09 | <.001** | 0.13 |  | 193.08 | <.001** | 0.06 |  |  |
| *Note.* All Chi-square tests degrees of freedom = 1; *χ²* = chi-square statistic; *df* = degrees of freedom *p* = significance level; * = significant at *p* <.05; ** = significant at *p* <.01; φ = phi (effect size). All comparisons significant after Bonferroni adjusted significance threshold (α = 0.006). PTSD = Post-Traumatic Stress Disorder; BPD = Borderline Personality Disorder; ADHD = Attention-Deficit Hyperactivity Disorder; OCD = Obsessive-Compulsive Disorder. | | | | | | | | | | |  |
|  | **Table 11d. Chi-Square Analysis of Comorbidities in Child Cohort - Male** | | | | | | | | | |  |
|  | |  | FND+Autism vs. FND-only | | |  | FND+Autism vs. Autism-only | | |  |  |
|  | |  | *χ²* | *p* | φ |  | *χ²* | *p* | φ |  |  |
| Mood disorders | |  | 70.93 | <.001** | 0.14 |  | 404.22 | <.001** | 0.05 |  |  |
| Anxiety disorders | |  | 89.99 | <.001** | 0.16 |  | 326.73 | <.001** | 0.04 |  |  |
| PTSD | |  | 2.89 | 0.08 | 0.03 |  | 147.79 | <.001** | 0.03 |  |  |
| Specific personality disorders | |  | 14.16 | <.001** | 0.06 |  | 8.823 | .002 | 0.01 |  |  |
| BPD | |  | 5.72 | .02^†^ | 0.04 |  | 3.93 | .05^†^ | 0.00 |  |  |
| Intellectual disabilities | |  | 165.34 | <.001** | 0.21 |  | 183.30 | <.001** | 0.08 |  |  |
| ADHD | |  | 330.49 | <.001** | 0.30 |  | 133.96 | <.001** | 0.03 |  |  |
| OCD | |  | 52.50 | <.001** | 0.12 |  | 58.10 | <.001** | 0.02 |  |  |
| *Note.* All Chi-square tests degrees of freedom = 1; *χ²* = chi-square statistic; *df* = degrees of freedom *p* = significance level; * = significant at *p* <.05; ** = significant at *p* <.01; φ = phi (effect size). ^†^ = not significant after Bonferroni adjusted significance threshold (α = 0.006). PTSD = Post-Traumatic Stress Disorder; BPD = Borderline Personality Disorder; ADHD = Attention-Deficit Hyperactivity Disorder; OCD = Obsessive-Compulsive Disorder. | | | | | | | | | | |  |

|  | **Table 12a. Odds ratios for Comorbidities in the Adult Cohort - Female** | | | | | | | | | | |
| --- | --- | --- | --- | --- | --- | --- | --- | --- | --- | --- | --- |
|  | |  | FND+Autism vs. FND-only | | |  | FND+Autism vs. Autism-only | | |  |  |
|  | |  | OR | 95% CI Lower | 95% CI Upper |  | OR | 95% CI Lower | 95% CI Upper |  |  |
| Mood disorders | |  | 3.13 | 2.78 | 3.53 |  | 5.49 | 4.88 | 6.20 |  |  |
| Anxiety disorders | |  | 3.25 | 2.88 | 3.67 |  | 4.73 | 4.19 | 5.34 |  |  |
| PTSD | |  | 2.06 | 1.88 | 2.27 |  | 5.89 | 5.36 | 6.49 |  |  |
| Specific personality disorders | |  | 2.89 | 2.61 | 3.21 |  | 3.89 | 3.50 | 4.32 |  |  |
| BPD | |  | 3.12 | 2.79 | 3.49 |  | 5.33 | 4.75 | 5.97 |  |  |
| Intellectual disabilities | |  | 12.85 | 11.27 | 14.67 |  | 1.42 | 1.25 | 1.60 |  |  |
| ADHD | |  | 5.19 | 4.73 | 5.69 |  | 2.28 | 2.08 | 2.50 |  |  |
| OCD | |  | 5.17 | 4.56 | 5.86 |  | 2.25 | 1.99 | 2.54 |  |  |
| *Note.* OR = Odds ratio; CI = Confidence Interval  PTSD = Post-Traumatic Stress Disorder; BPD = Borderline Personality Disorder; ADHD = Attention-Deficit Hyperactivity Disorder; OCD = Obsessive-Compulsive Disorder. | | | | | | | | | | |  |
|  | **Table 12b. Odds ratios for Comorbidities in the Adult Cohort - Male** | | | | | | | | | |  |
|  | |  | FND+Autism vs. FND-only | | |  | FND+Autism vs. Autism-only | | |  |  |
|  | |  | OR | 95% CI Lower | 95% CI Upper |  | OR | 95% CI Lower | 95% CI Upper |  |  |
| Mood disorders | |  | 2.50 | 2.21 | 2.83 |  | 5.05 | 4.48 | 5.69 |  |  |
| Anxiety disorders | |  | 2.94 | 2.60 | 3.33 |  | 4.70 | 4.15 | 5.31 |  |  |
| PTSD | |  | 1.57 | 1.37 | 1.80 |  | 7.16 | 6.26 | 8.20 |  |  |
| Specific personality disorders | |  | 2.50 | 2.14 | 2.92 |  | 4.61 | 3.97 | 5.36 |  |  |
| BPD | |  | 3.29 | 2.74 | 3.97 |  | 4.92 | 4.12 | 5.87 |  |  |
| Intellectual disabilities | |  | 8.03 | 6.92 | 9.31 |  | 1.52 | 1.33 | 1.73 |  |  |
| ADHD | |  | 6.61 | 5.88 | 7.42 |  | 1.85 | 1.66 | 2.07 |  |  |
| OCD | |  | 6.57 | 5.58 | 7.73 |  | 2.52 | 2.17 | 2.92 |  |  |
| *Note.* OR = Odds ratio; CI = Confidence Interval  PTSD = Post-Traumatic Stress Disorder; BPD = Borderline Personality Disorder; ADHD = Attention-Deficit Hyperactivity Disorder; OCD = Obsessive-Compulsive Disorder. | | | | | | | | | | |  |

|  | **Table 12c. Odds ratios for Comorbidities in the Child Cohort - Female** | | | | | | | | | | |
| --- | --- | --- | --- | --- | --- | --- | --- | --- | --- | --- | --- |
|  | |  | FND+Autism vs. FND-only | | |  | FND+Autism vs. Autism-only | | |  |  |
|  | |  | OR | 95% CI Lower | 95% CI Upper |  | OR | 95% CI Lower | 95% CI Upper |  |  |
| Mood disorders | |  | 3.01 | 2.53 | 3.59 |  | 6.59 | 5.55 | 7.83 |  |  |
| Anxiety disorders | |  | 3.05 | 2.49 | 3.74 |  | 6.94 | 5.68 | 8.47 |  |  |
| PTSD | |  | 1.75 | 1.40 | 2.19 |  | 7.51 | 6.03 | 9.35 |  |  |
| Specific personality disorders | |  | 3.59 | 2.53 | 5.10 |  | 4.79 | 3.46 | 6.63 |  |  |
| BPD | |  | 4.11 | 2.72 | 6.21 |  | 5.98 | 4.10 | 8.73 |  |  |
| Intellectual disabilities | |  | 12.30 | 9.10 | 16.62 |  | 1.97 | 1.52 | 2.57 |  |  |
| ADHD | |  | 3.47 | 2.98 | 4.05 |  | 1.98 | 1.72 | 2.29 |  |  |
| OCD | |  | 4.06 | 3.18 | 5.20 |  | 4.41 | 3.51 | 5.53 |  |  |
| *Note.* OR = Odds ratio; CI = Confidence Interval  PTSD = Post-Traumatic Stress Disorder; BPD = Borderline Personality Disorder; ADHD = Attention-Deficit Hyperactivity Disorder; OCD = Obsessive-Compulsive Disorder. | | | | | | | | | | |  |
|  | **Table 12d. Odds ratios for Comorbidities in the Child Cohort - Male** | | | | | | | | | |  |
|  | |  | FND+Autism vs. FND-only | | |  | FND+Autism vs. Autism-only | | |  |  |
|  | |  | OR | 95% CI Lower | 95% CI Upper |  | OR | 95% CI Lower | 95% CI Upper |  |  |
| Mood disorders | |  | 2.29 | 1.88 | 2.78 |  | 5.14 | 4.30 | 6.14 |  |  |
| Anxiety disorders | |  | 2.46 | 2.04 | 2.97 |  | 4.39 | 3.69 | 5.23 |  |  |
| PTSD | |  | 1.34 | 0.97 | 1.85 |  | 5.28 | 3.93 | 7.10 |  |  |
| Specific personality disorders | |  | 3.09 | 1.72 | 5.56 |  | 2.14 | 1.32 | 3.47 |  |  |
| BPD | |  | 2.63 | 1.25 | 5.52 |  | 2.00 | 1.06 | 3.75 |  |  |
| Intellectual disabilities | |  | 5.60 | 4.20 | 7.46 |  | 2.38 | 1.90 | 2.98 |  |  |
| ADHD | |  | 5.61 | 4.59 | 6.85 |  | 2.83 | 2.36 | 3.41 |  |  |
| OCD | |  | 3.69 | 2.55 | 5.34 |  | 3.09 | 2.29 | 4.18 |  |  |
| *Note.* OR = Odds ratio; CI = Confidence Interval  PTSD = Post-Traumatic Stress Disorder; BPD = Borderline Personality Disorder; ADHD = Attention-Deficit Hyperactivity Disorder; OCD = Obsessive-Compulsive Disorder. | | | | | | | | | | |  |
